# An early surge of norepinephrine along brainstem pathways drives sensory-evoked awakening

**DOI:** 10.1126/sciadv.adw6375

**Published:** 2025-09-10

**Authors:** Noa Matosevich, Noa Regev, Uddi Kimchy, Noam Zelinger, Sina Kabaha, Noam Gabay, Amit Marmelshtein, Yuval Nir

**Affiliations:** ^1^ Sagol School of Neuroscience, Tel Aviv University, Tel Aviv, Israel.; ^2^ Department of Physiology and Pharmacology, Gray Faculty of Medical and Health Sciences, Tel Aviv University, Tel Aviv, Israel.; ^3^ Institute for Physiology, Faculty of Medicine, University of Freiburg, 79108 Freiburg, Germany.; ^4^ Sagol Brain Institute, Tel Aviv Sourasky Medical Center, Tel Aviv, Israel.; ^5^ Department of Biomedical Engineering, Faculty of Engineering, Tel Aviv University, Tel Aviv, Israel.; ^6^ The Sieratzki-Sagol Center for Sleep Medicine, Tel Aviv Sourasky Medical Center, Tel Aviv, Israel.

## Abstract

The locus coeruleus–norepinephrine (LC-NE) system regulates arousal and awakening; however, it remains unclear whether the LC does this in a global or circuit-specific manner. We hypothesized that sensory-evoked awakenings are predominantly regulated by specific LC-NE efferent pathways. Anatomical, physiological, and functional modularities of LC-NE pathways involving the mouse basal forebrain (BF) and pontine reticular nucleus (PRN) were tested. We found partial anatomical segregation between the *LC → PRN* and *LC → BF* circuits. Extracellular NE dynamics in BF and PRN exhibited distinct sound-evoked activation during sleep, including a fast sound-evoked NE peak specific to PRN. Causal optogenetic interrogation of LC efferent pathways, by retro-channelrhodopsin (ChR2) activation or Platynereis dumerilii ciliary opsin (PdCO) silencing of synapses in target regions, revealed a role for early *LC → PRN* activity in driving arousal and sound-evoked awakenings. Together, our results uncover a role for early LC-NE *PRN* activity in connecting sensory and arousal pathways and establish LC heterogeneity in regulating arousal.

## Teaser

The locus coeruleus has a specialized pathway to the brainstem that controls sound-evoked awakening from sleep.

## INTRODUCTION

Engagement with the environment is critical to our survival as it allows us to perceive changes, respond to cues, and adapt behavior to diverse situations including potential threats. Sleep is dominated by reduced responsiveness to the environment, rendering animals vulnerable to predation. An elevated “arousal threshold” characterizes sleep across all species and constitutes the main criterion by which sleep is defined in invertebrates lacking cortical electroencephalogram (EEG) (*1*–*5*), yet the pathways underlying the neural basis of sensory disconnection during sleep are not fully understood (*6*–*9*).

An important neuromodulatory element in shaping responsiveness to external sensory events across internal states is locus coeruleus–norepinephrine (LC-NE) activity. Accordingly, LC-NE activity correlates with sleep fragility and microarousals and is inversely related to spindle activity associated with sleep continuity (*9*–*11*). In wakefulness, LC-NE activity supports orienting responses toward behaviorally meaningful salient stimuli (*12*–*16*). Moreover, LC-NE activity was recently shown to be a major determinant of sound-evoked awakening (SEA) in rats (*17*). LC activation around auditory stimulation in sleep increased the probability of waking up in response to a sound, whereas LC silencing showed the inverse effect.

However, while LC-NE neuromodulation was traditionally regarded as globally homogenous (*18*–*23*), recent studies identified some segregation of efferent LC pathways along with associated functional diversity [reviewed in (*24*)]. For example, spinally projecting LC neurons exert analgesic actions, whereas ascending LC projections show a pronociceptive effect (*25*–*27*). LC neurons projecting to the prefrontal versus motor cortex differ in their molecular profiles, excitability, and activity across vigilance states (*28*). Furthermore, LC neuronal firing has been shown to be only sparsely synchronized (*29*). This raises the following question: Could heterogenous LC-NE activity also play a role within the domain of arousal and sleep awakenings?

To address this, we investigated whether the effect of LC on SEA is mediated by distinct LC projections. We focused on two target regions, the pontine reticular nucleus (PRN) and the basal forebrain (BF), for representing brainstem and forebrain target regions, respectively, as both areas are profoundly innervated by LC-NE neurons and are implicated in regulating arousal (*30*–*35*). We combined anatomical mapping of PRN- or BF-projecting neurons, GRAB_NE_-based extracellular NE recordings at these target regions, and bidirectional optogenetic manipulations of presynaptic LC terminals in these projections (*36*). Our results reveal a specific role for an early surge of brainstem NE in driving SEA from sleep.

## RESULTS

### LC neurons projecting to the BF and PRN are partially distinct anatomically

**Fig. 1.**
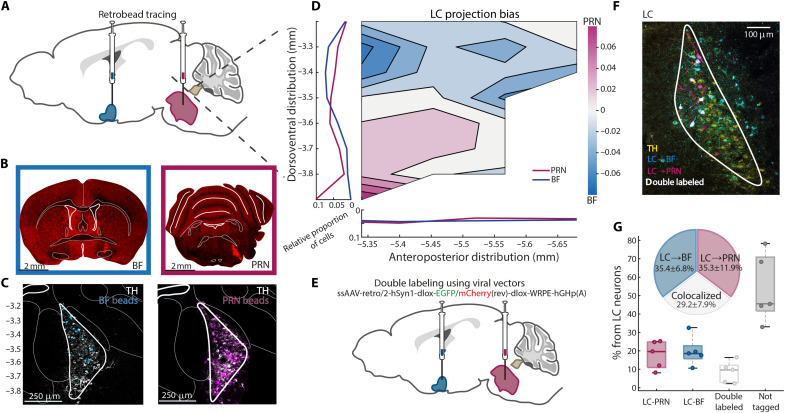
LC neurons projecting to the BF and PRN are partially distinct anatomically. (**A**) Surgical approach: Sagittal section taken from the Allen Mouse Brain Atlas. (**B**) Representative examples of BF retrobead injection (left) and PRN retrobead injection (right). (**C**) The LC section at −5.4 mm from bregma (AP). BF bead tracing in blue (left), PRN bead tracing in magenta (right), and tyrosine hydroxylase (TH) staining in white. A mask was applied to highlight the LC region since retrobeads are not specific in their tagging. (**D**) Normalized projection pattern of LC neurons to *BF* or *PRN* calculated as $\frac{{\text{LC}}_{\text{PRN}}-{\text{LC}}_{\text{BF}}}{\text{Total}}$ calculated over *n* = 8 PRN injected mice and *n* = 7 BF injected mice. Blue tones on the heatmap represent a higher probability of *BF-projecting cells*, and magenta tones represent a higher probability of *PRN-projecting cells*. The image portrays the LC in a sagittal section. To the left are the average traces of the LC cell distribution on the DV axis from the top corner of the LC (−3.2 mm relative to bregma) to −3.9 mm from bregma. At the bottom are the average distributions of the LC neurons on the AP axis from −5.34 to −5.7 mm from bregma. Sections were identified based on the Allen Mouse Brain Atlas (*68*), and, thus, coordinates were determined. (**E**) Sagittal section taken from the Allen Mouse Brain Atlas to portray the approach. (**F**) LC section of representative mouse. TH, yellow; *LC → BF*, blue; *LC → PRN*, magenta; white, colocalized cells. (**G**) Box plot of LC cells divided to neurons projecting to *PRN* only, *BF* only, both *PRN and BF*, or *neither* (gray). Circles represent individual mice. Inlaid is a pie chart of colocalization percentage of the tagged cells (excluding neurons that were not tagged by either virus). Average and SD calculated over *n* = 5 DBH-Cre mice.

We first investigated whether LC cells projecting to the BF and those projecting to the PRN exhibit anatomical segregation. Using retrograde tracing (Fig. 1A), retrobeads were injected either in the BF or the PRN (*n* = 7 and *n* = 8, respectively) of wild-type (WT) mice, and successful targeting was histologically confirmed (Fig. 1B and table S1). LC subpopulation analysis (Fig. 1C) determined the distribution of LC neurons along the dorsoventral (DV) and the anteroposterior (AP) axes of the LC (Fig. 1D). We found a statistically significant difference in the anatomical distribution of *BF-projecting* versus *PRN-projecting* LC neurons (*P* = 0.004, Monte Carlo permutation test), implying that the *BF* and *PRN* neuronal projections of the LC are different subpopulations. Specifically, *BF-projecting LC neurons* were localized more dorsally in the LC compared to the *PRN-projecting LC neurons* (*P* = 6 × 10^−4^, Monte Carlo permutation test). No projection bias was observed along the AP axis (*P* = 0.4, Monte Carlo permutation test).

Next, to determine the degree of cellular colocalization among the two subpopulations, we conducted retrograde viral tracing by performing simultaneous injections of red and green Cre-dependent AAVretro viruses into both PRN and BF [*n* = 5 dopamine beta-hydroxylase (DBH)–Cre mice; Fig. 1, E and F]. The quantitative analysis revealed that 53.9 ± 18.7% of LC cells were not tagged by any virus. The tagged LC neurons consisted of 35.34 ± 11.95% *PRN-only* projecting cells, 35.44 ± 6.79% *BF-only* projecting cells, and 29.21 ± 7.93% *colocalized* cells (projecting to both target regions), meaning that the LC contains both distinct subpopulation as well as neurons sending axonal collaterals to both *BF and PRN* (Fig. 1G and table S4). *t* tests against the null hypothesis of homogeneity was significant, confirming that each subpopulation is distinct compared to the overlapping subpopulation and that neither *LC → PRN* nor *LC → BF* is a subpopulation of one another [*LC → PRN*: *P* = 0.0041, *t*_4_ = 5.91; *LC → BF*: *P* = 4.669 × 10^−4^, *t*_4_ = 10.49]. Thus, beyond a DV gradient in the distribution of *BF-projecting LC cells*, most tagged LC neurons were not labeled by both viruses and, thus, project to one of the target regions, either to *PRN* or to *BF* but not to both. The results establish partial anatomical modularity in brainstem versus forebrain LC projections.

### A fast brainstem-specific auditory-evoked NE response predicts awakening

Next, we compared extracellular NE dynamics between brainstem and forebrain target regions with GRAB_NE_ (*37*). We first validated our GRAB_NE_ tool by testing BF GRAB_NE_ dynamics in response to optogenetic LC stimulation (fig. S1). Results revealed a dose-dependent increase in pupil dilation and NE levels with stronger optogenetic LC stimulation in lightly anesthetized mice as in (*17*). In addition, induced sleep-to-wake transitions in freely behaving mice were associated with elevated NE levels and EEG activation (*38*), confirming that GRAB_NE_ records NE dynamics that are controlled by LC neurons.

**Fig. 2.**
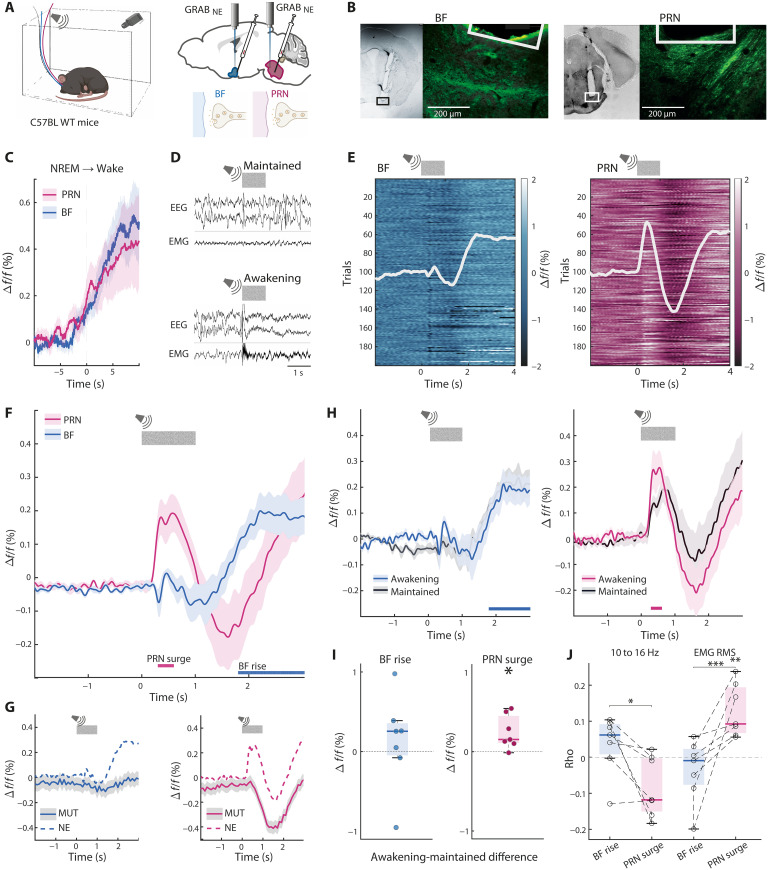
A fast brainstem-specific auditory-evoked NE response predicts awakening. (**A**) Depiction of experimental setup. Created in BioRender. N. REGEV (2025); https://BioRender.com/2zvhz0k. (**B**) Representative examples of fiber-optic and GRAB_NE_ expression in BF (left) and PRN (right). (**C**) Average GRAB_NE_ traces in BF (blue) and PRN (magenta) during spontaneous awakening from NREM sleep across mice (*n* = 7); *P* = 0.53, *t*_6_ = 0.66. (**D**) Examples of trials that led to maintained sleep (top) or awakening (bottom). (**E**) All NREM SEA trials in a representative mouse. Left: BF; right: PRN. White graph overlaid represents the average trace. (**F**) Average NREM SEA traces in BF (blue) and PRN (magenta) across mice. Horizontal lines represent time periods that are significantly above 0 for PRN and BF. (**G**) As a control group, GRAB_MUT_ experiments were run on *n* = 4 mice each in BF (blue) and PRN (magenta). Experiments were conducted on mice with only one injection site and fiber placement, either BF or PRN. Data were averaged across all trials forgoing scoring, using the same pipeline as for GRAB_NE_. Dashed lines represent the corresponding traces of GRAB_NE_. (**H**) Average traces across mice of maintained and awakening trials. BF: left, blue; PRN: right, magenta. Horizontal lines indicate BF rise and PRN surge, respectively. (**I**) Box plots of signal differences between trials with SEA and maintained sleep during BF rise and PRN surge. Left: BF rise; right: PRN surge. Dots represent single animals. PRN surge: *P* = 0.02, *t*(6) = −3.06; BF rise: *P* = 0.58, *t*_6_ = −0.58. (**J**) Correlation values between mean EEG and EMG and response elements (BF rise, blue; PRN surge, magenta). EEG 10 to 16 Hz band (left): *P* = 0.013, *F*_2_ = 5.58; *P* = 0.01, post hoc PRN surge versus BF rise; EMG RMS (right): *P* = 4 × 10^−4^, *F*_2_ = 12.39; *P* = 4.4 × 10^−3^, PRN surge versus zero; *P* = 5 × 10^−4^, PRN surge versus BF rise. *P < 0.05; **P < 0.01; ***P < 0.001.

To directly compare between NE dynamics in the BF and PRN, we injected WT mice (*n* = 7) with GRAB_NE_ in both BF and PRN and recorded fiber photometry simultaneously from the two areas during natural sleep (Fig. 2, A and B). Around spontaneous awakenings from nonrapid eye movement (NREM) sleep (fig. S2), both target regions exhibited similar dynamics of increased NE levels with no discernable difference between the two (Fig. 2C). By contrast, SEA experiments (Fig. 2D) revealed distinct NE dynamics in the PRN compared with the BF (Fig. 2, E and F). While the PRN NE dynamics exhibited a fast surge in response to sounds during sleep, BF NE showed a slower, later response.

Time series analysis across mice (methods; Fig. 2F) revealed a time window in the PRN NE trace where activity was significantly different than baseline: “PRN surge” (*time* = 0.3 to 0.6 s; *P* = 0.035). Following this component, a signal drop in GRAB_NE_ was significantly correlated with the preceding positive peak (Pearson’s *R* = −0.52; *P* = 1.03 × 10^−100^). Similar analysis in the BF NE traces revealed a different time window with significant activity: “BF rise” (*time* = 1.8 to 3 s; *P* = 0.015, 0.004, 0.005, and 0.01).

Experiments with a mutated GRAB construct as a control (GRAB_MUT_) did not reveal an early positive peak, confirming that the early component represents NE dynamics rather than an artifact (Fig. 2G). However, GRAB_MUT_ dynamics in the PRN also revealed a signal drop ~1.5 s after sound onset. This signal was not significantly associated with behavioral trial outcome [i.e., awakening versus maintained sleep; *t*_3_ = 1.56, *P* = 0.21]. Furthermore, GRAB_MUT_ results exhibited high variability across mice and no substantial correlations with spectral dynamics in the EEG delta (<4 Hz) or sigma (10 to 16 Hz) ranges (Rho = 0.02 ± 0.1 and 0.01 ± 0.05, respectively; means ± SD) or with the root mean square (RMS) of the electromyogram (EMG) muscle tone (Rho = −0.03 ± 0.06).

Next, we examined which response element was associated with behavioral trial outcome (awakening versus maintained sleep; Fig. 2, H and I). The early brainstem NE response (PRN surge) was elevated in trials in which the sound elicited awakening compared to trials in which the mouse maintained sleep after sound, implying its unique importance to SEA. Next, we applied a multinomial regression model to our data, using each of the elements (BF rise and PRN surge) to predict behavioral outcome in each trial. This model proved to be highly significant (χ^2^ compared to the constant model: 17.51, *P* = 1.6 × 10^−4^; table S6). The PRN response was the strongest determining factor.

To examine the relationship between GRAB_NE_ and EEG/EMG dynamics above and beyond the effect on awakening, we calculated the correlation coefficients between response elements (BF rise and PRN surge) and EEG bands/EMG RMS for each mouse. Specifically, power in the EEG sigma band (10 to 16 Hz) reflects sleep spindle activity (*39*), which has been implicated in plasticity and disconnection from the environment (*40*), and so, was of interest to test the relation of GRAB_NE_ dynamics to such activity. Significant differences in correlation with the EEG sigma band were found between PRN surge and BF rise (Fig. 2J). Correlations with EMG RMS were also significantly different, with PRN surge correlating positively and significantly above zero. These findings show that the PRN surge is uniquely anticorrelated with sleep spindle activity and positively correlated with EMG amplitude, showing a trial-by-trial connection to physiological signals related to behavioral engagement with the environment.

To determine whether the fast PRN NE activation stems from LC neuronal activity or whether it is driven by dynamics at the target brainstem synapse, we recorded GCaMP7s from LC subpopulations projecting to the PRN. DBH-Cre mice were injected with AAVretro vectors encoding a Cre-dependent GCaMP7s in the PRN and BF (*n*_PRN_ = 7, *n*_BF_ = 7), and bulk calcium activity was recorded in the LC corresponding subpopulation (fig. S3, A and B). We found that the fast sound-evoked activity was already present to some extent in the activity of *LC → PRN* projecting neurons (fig. S3D), suggesting that the rapid NE signal measured in PRN during SEA is partially explained by upstream LC activity. To directly compare LC versus target region NE dynamics, we overlaid the previously recorded GRAB_NE_ traces with the GCaMP7s signals (fig. S3, C and D). Cross-correlation analysis (fig. S3E) revealed strong correlations during awakening trials, particularly aligning with the slower rise components of the NE signal (peak correlation at *time* = 1.84 s for BF and *time* = 2.6 s for PRN). Notably, in awakening trials, but not in trials with maintained sleep, the cross-correlation exhibited a distinct peak for BF and a local peak at *time* = 0 for PRN, corresponding with the PRN NE surge. Furthermore, when comparing mean calcium responses over the full 3 s following stimulus onset, we observed a significant difference between maintained and awakening trials for *LC → PRN*, but not *LC → BF* (fig. S3F).

### Early LC → PRN surge drives awakening

**Fig. 3.**
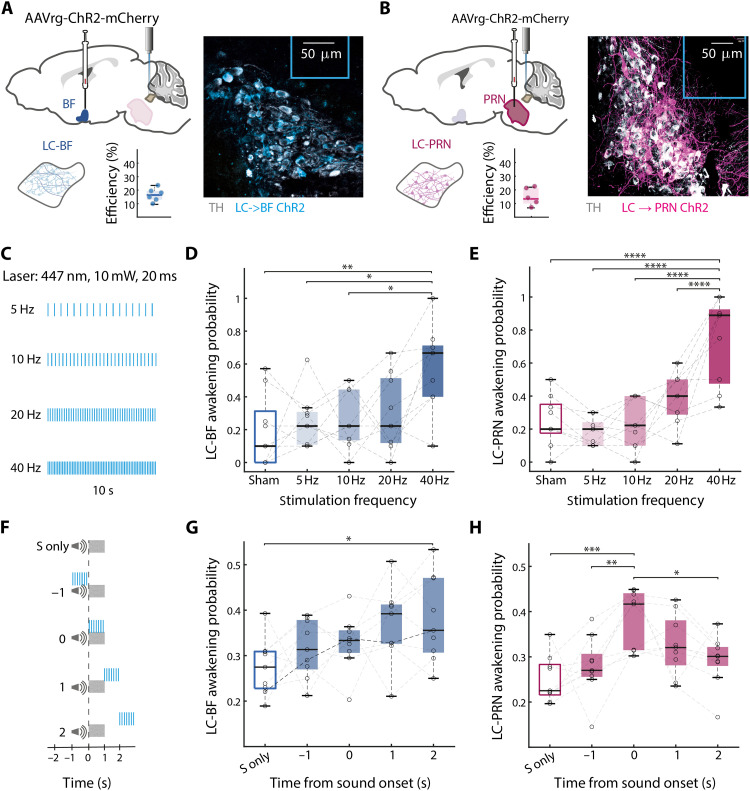
Early LC → PRN surge drives awakening. (**A**) Depiction of surgical approach for *LC → BF* and average viral efficiency (left), and representative examples of fiber-optic(cerulean line) and ChR2 expression in *LC → BF* (right). White, TH; blue, viral expression. (**B**) Depiction of surgical approach for *LC → PRN* as well as average viral efficiency (left), and representative examples of fiber-optic (fiber-optic line) and ChR2 expression in *LC → PRN* (right). White, TH; magenta, viral expression. (**C**) Experimental procedure for laser awakening experiment. The experiment included 10-s laser of 10 mW and 20-ms duty cycle for 5, 10, 20, and 40 Hz. (**D** and **E**) Box plot of probability to awaken from LC laser activation as a function of frequency in *LC → BF* ChR2 [*P* = 0.004, *F*_4_ = 4.6; sham versus 40 Hz: *P* = 0.004, 5 versus 40 Hz: *P* = 0.02, and 10 versus 40 Hz: *P* = 0.016] (D) and *LC → PRN* ChR2 [*P* = 5.85 × 10^−8^, *F*_4_ = 16.18; post hoc: sham versus 40 Hz: *P* = 2.8 × 10^−6^, 5 versus 40 Hz: *P* = 2.06 × 10^−7^, 10 versus 40 Hz: *P* = 1.11 × 10^−6^, and 20 versus 40 Hz: *P* = 6.11 × 10^−4^] (E). Dots represent single animals (*n*_BF_ = 9, *n*_PRN_ = 9). (**F**) Experimental procedure for timing experiment. The experiment included either sound only (S only) or 1-s laser of 10 mW and 20-ms duty cycle, 20 Hz combined with a BBN in 80 dB SPL, so that the laser activation happened in different intervals relative to the sound: 1 s before, on time, 1 s after, or 2 s after. (**G** and **H**) Box plot of probability to awaken from LC laser activation as a function of timing in *LC → BF* ChR2 [*P* = 0.029, *F*_4_ = 3.01; sound only versus 2 s: *P* = 0.032] (G) and *LC → PRN* ChR2 [*P* = 0.0009, *F*_4_ = 5.77; sound only versus on time: *P* = 0.0007, 1 s before versus on time: *P* = 0.01, and on time versus 1 s after: *P* = 0.033, and on time versus 2 s after: *P* = 0.02] (H). Dots represent single animals (*n*_BF_ = 9, *n*_PRN_ = 9). *P < 0.05; **P < 0.01; ***P < 0.001; ****P < 1 × 10^−4^.

To determine whether *LC → BF* and *LC → PRN* increased activity are sufficient to cause awakening and SEA, we injected Cre-dependent AAVretro vector with ChR2, an excitatory opsin, to either BF (*n* = 9) or PRN (*n* = 9) and placed an fiber-optic above the LC to induce specific activation of LC subpopulations (Fig. 3, A and B, and table S5). First, we checked whether 10 s of continuous optogenetic activation of each subpopulation promotes awakening (Fig. 3C). In line with our results from GRAB_NE_ recordings, indicating similar activation in spontaneous awakening, in this experiment, we found that both subpopulations are sufficient to induce awakening. We observed that activating the *LC → PRN* subpopulation induces awakening in a frequency-dependent manner (Fig. 3E), with 40-Hz stimulation being significantly more effective than lower stimulation frequencies. *LC → BF* activation similarly depended on stimulation frequency (Fig. 3D).

Next, we investigated the effect of optogenetically activating *LC → PRN* and *LC → BF* during sleep when combined with sound presentation. Activation of 20 Hz occurred for 1 s either before sound onset, together with sound presentation, or after sound presentation (Fig. 3F). Given the distinct early peak in PRN NE and its relationship with SEA, we hypothesized that *LC → PRN* effects on awakening would depend on stimulation occurring together with sound, whereas *LC → BF* would be more strongly affected when stimulation occurs after sound offset. In line with this prediction, *LC → PRN* activation showed a strong effect on awakening probability when laser stimulation was synchronized with sound (Fig. 3H). Furthermore, the effects of *LC → BF* optogenetic activation on awakening were associated with timing relative to auditory stimulation so that only stimulation of *BF-projecting LC neurons* 2 s after sound onset increased awakening probability (Fig. 3G).

Together with fluorophore-only control that shows no effect (fig. S4), these results demonstrate that both subpopulations have a frequency and time dependency. The differences in the crucial timings of stimulation between the *LC → BF* and *LC → PRN* demonstrate the differences in these LC subpopulations. Furthermore, the timing of *LC → PRN* activation implies coherence and a more direct involvement in sensory processing, whereas the later *LC → BF* activation is more in line with the timing of the state changes than of the stimuli.

### Silencing the LC → PRN pathway strengthens EEG sigma power during NREM sleep

**Fig. 4.**
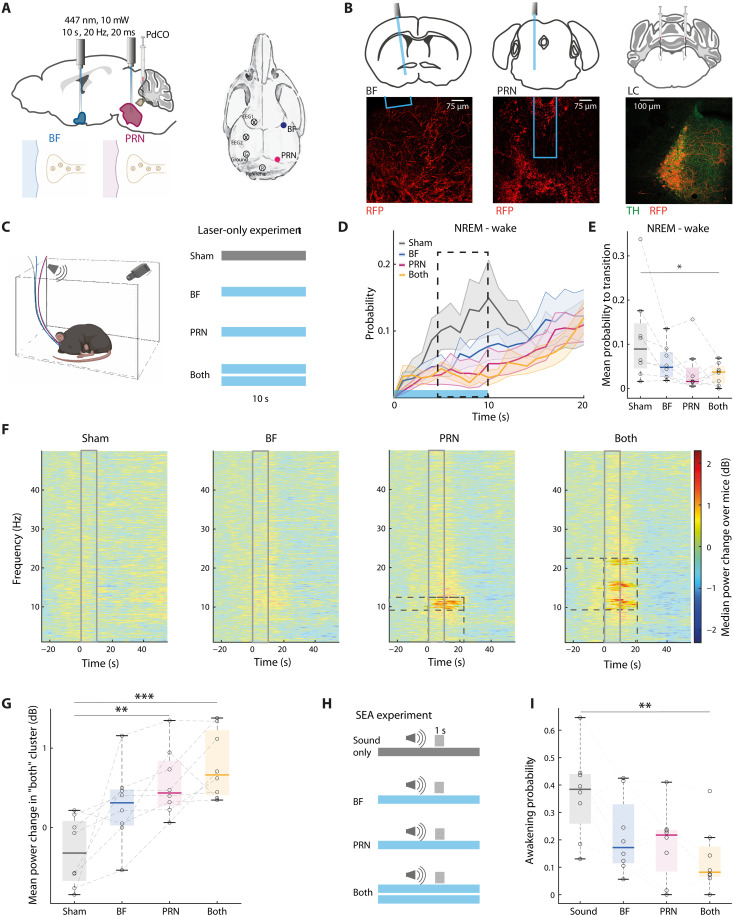
Silencing the LC→PRN pathway strengthens EEG sigma power during NREM sleep. (**A**) Depiction of surgical approach. (**B**) Representative examples of fiber and PdCO expression (RFP) in BF (left), PRN (middle), and LC (right). Green, TH; red, viral expression. (**C**) Left: Experimental setup [Created in BioRender. N. REGEV (2025); https://BioRender.com/2zvhz0k]; right: experimental procedure. The experiment included 10-s laser of 10 mW, 20-ms duty cycle, 20-Hz stimulation activated in *BF*, *PRN*, *both*, and sham trials. (**D**) Probability to transition from NREM to wake. Gray, sham; blue, *BF*; magenta, *PRN*; yellow, *both*. Laser was on from *time* = 0 to 10 s, depicted by cerulean line in bottom. Dashed square represents the time taken for further analysis (*time* = 5 to 10 s). (**E**) Box plot of average probability to transition from NREM to wake under each condition during *time* = 5 to 10 from laser onset as marked in panel (D). *P* = 0.05, *F*(3) = 2.94; sham versus *both*: *P* = 0.05; all other comparisons not significant. (**F**) Median EEG spectrograms across mice around trials during NREM normalized to *time* = −30 to 0 s relative to laser onset. Laser was on *time* = 0 to 10 s. From left to right: sham, *BF* laser, *PRN* laser, and *both* lasers on. Areas that are significant are fully colored (rather than opaque) and have dashed squares around them. Cluster statistics: *P*_BF_ = 0.42, *P*_PRN_ = 0.04, *P*_both_ = 0.03. (**G**) Average mean power change within the significant cluster found in “both” panel (F) under each of the conditions. Gray, sham; blue, *BF*; magenta, *PRN*; yellow, *both*. *P* = 2 × 10^−4^, *F*_3_ = 9.16; sham versus *BF*: *P* = 0.06, sham versus *PRN*: *P* = 0.003, and sham versus *both*: *P* = 2 × 10^−4^. (**H**) Experimental procedure. The experiment included 10-s laser of 10 mW, 20-ms duty cycle, 20-Hz stimulation activated in *BF*, *PRN*, *both* simultaneously, and sham trials. At *time* = 8 s, BBN of 80 dB SPL was played by a mounted speaker for 1 s. (**I**) Box plot of awakening probability. *P* = 0.012, *F*_3_ = 4.4; *both* versus sound: *P* = 0.008, *PRN* versus sound: *P* = 0.06, and *BF* versus sound: *P* = 0.14; Tukey-Kramer post hoc tests. *P < 0.05; **P < 0.01; ***P < 0.001.

Last, we set out to investigate whether NE release along specific projection pathways is necessary for effects on EEG and on awakening probability. To this end, we selectively silenced synaptic release of LC neurons in either BF or PRN target regions using the inhibitory optoGPCR PdCO (*41*) (*n* = 8 DBH-Cre mice). We injected a Cre-dependent PdCO viral construct to the LC and placed fiber-optics above both the PRN and BF target regions (Fig. 4, A to C). While PdCO can be used as a switchable optoGPCR, using 477-nm blue light, as done here, effectively leads to short-lasting silencing of synaptic release, as shown previously (*41*). We first examined the effect of silencing on otherwise uninterrupted sleep to analyze spontaneous awakening. Silencing LC synapses simultaneously in both areas consolidated NREM sleep, as evident by lower probability to transition to wakefulness (Fig. 4, D and E). Furthermore, silencing *LC → PRN* synapses alone during NREM sleep significantly elevated EEG power in the low frequencies of the sigma band (9.5 to 12.25 Hz), while silencing both *PRN* and *BF* pathways simultaneously additionally led to a power increase in the higher end of the sigma band (14.2 to 16.8 Hz), together with increase in the power of 20.7 to 22.35 Hz (Fig. 4F and fig. S5). A comparison of average power in the combined cluster confirmed that *PRN* silencing, as well as silencing in both targets, significantly elevated power in the EEG (Fig. 4G). Previous studies established anticorrelation between LC activity and sigma activity (reflecting sleep spindles) (*9*, *42*–*44*), associated with more resilient sleep, and reduced arousal measures such as heart rate and pupil size (*8*, *45*). Thus, silencing *LC → PRN* synapses and the resultant elevated sigma power reflect more consolidated sleep (*46*).

Next, we examined the effect of silencing LC synapses in target regions with respect to SEA (−8 + 2 s around sound onset; Fig. 4H). Such silencing lowered the probability to awaken by external stimulus (Fig. 4I). Evoked awakening probability, when simultaneously silencing both target regions, was significantly lower than sham. *PRN* silencing showed a trend (*P* = 0.06, Tukey-Kramer post hoc test) for reduced awakening probability, while silencing the *BF* alone was not significantly different compared to sham stimulation (*P* = 0.14, Tukey-Kramer post hoc test). We found no significant difference in arousal probability between *BF* and *PRN*. Fluorophore-only control experiments confirmed that laser application itself was not associated with significant changes in awakening probability (fig. S6).

Together, these projection-specific silencing experiments show that inhibiting LC synaptic release in both the *PRN* and *BF* reduces awakening probability and alters EEG signatures during sleep. While suppression of either target alone had a limited effect on arousal probability, silencing both significantly reduced sound-evoked and spontaneous awakenings. Silencing the *PRN* alone was sufficient to enhance sigma power in the EEG, suggesting that this projection may have a particularly prominent role in modulating sleep-related neural dynamics.

### Early PRN spiking responses to sounds are modulated by LC → PRN input

Last, to test whether PRN spiking activities exhibit early responses modulated by *LC → PRN*, in line with the fast NE signal kinetics in that region, we performed PRN electrophysiological recordings using a 16-channel optoelectrode (NeuroNexus) in lightly anesthetized (0.9% isoflurane) DBH-Cre mice, injected three weeks before with a Cre-dependent AAVretro-ChR2 virus for optogenetic tagging (fig. S7A). Based on our results in SEA experiments and on research from other groups (*47*, *48*), we hypothesized that a substantial proportion of PRN neurons targeted by LC may be auditory responsive. To test this, we combined optogenetic and auditory stimulation to identify sound-responsive PRN neurons that likely receive monosynaptic input from LC fibers (fig. S7, B to G).

We identified *n* = 49 neurons across three recorded mice. Of those, 69.4% responded to optogenetic stimulation of LC fibers, 46.9% responded to auditory stimuli, and 24.5% responded to neither. Notably, 40.8% of the units responded to both auditory and optostimulation (fig. S7C). This corresponded to the majority of the auditory-responsive PRN neurons (86.9%) and over half of the optotagged neurons (58.8%). The auditory response was observed within 20 ms from sound onset (fig. S7D), in line with the fast NE kinetics we observed in LC → PRN pathways during SEAs.

## DISCUSSION

We investigated whether neuronal and behavioral responses to sounds during sleep recruit the LC-NE system globally or via projection-specific pathways. To this end, we examined arousal-promoting LC-NE pathways involving the brainstem (*PRN*) and the *BF*. Our results establish that these LC-NE pathways differentially contribute to SEA during sleep, with an early surge of activity in the *LC → PRN* pathway playing a privileged role. We show that LC-NE heterogeneity arises both in terms of the LC neurons (when monitoring or manipulating specific subpopulations via AAVretro vectors) as well as through NE dynamics in target regions (when monitoring extracellular NE using GRAB_NE_ or manipulating synaptic release via PdCO). Together, the process of SEA is modulated by an early activity of *LC → PRN* projecting neurons and NE release and dynamics at the brainstem, attesting to modularity in LC-NE signaling even in the context of sleep and arousal.

Anatomically, LC neurons targeting the forebrain are located more dorsally compared to those projecting to the PRN (Fig. 1). This dorsal-to-ventral gradient is reminiscent of past anatomical studies that indicate that the LC is organized by projection target (*26*, *49*). Anatomical distinction was further reflected physiologically (Fig. 2), with sound-evoked NE activity patterns differing significantly between the PRN and the BF. Specifically, NE profile at the PRN exhibited a strong rapid surge of activity that was tightly synchronized with the sensory stimulus and predicted whether the animal would awaken or remain asleep within seconds. Optogenetic manipulation (Fig. 3) established the causal role of rapid NE surges in the *LC → PRN* pathway during SEA. Inducing this surge concurrently with auditory stimuli significantly increased awakening probability. Moreover, silencing *LC → PRN* synaptic release (Fig. 4) exhibited some signs of consolidating NREM sleep to a greater extent than *LC → BF* silencing, further supporting the notion that the *LC → PRN* pathway is important in promoting awakening.

These findings highlight multilayered heterogeneity within the LC-NE system, promoting the view that arousal signaling is circuit-specific rather than globally uniform. Accordingly, LC-NE modularity is evident both in NE dynamics at target regions (as evident when using GRAB_NE_ and PdCO) and in activities of presynaptic LC subpopulations based on their projections (as evident using AAVretro vectors with GCaMP7s and ChR2). Thus, heterogeneity in the LC-NE system requires investigation of several complementary mechanisms occurring simultaneously presynaptically, at the synapse, at postsynaptic receptors, and at non-neuronal compartments (*20*, *50*, *51*). Our results join previous work that showed LC modularity in the context of other functions such as anxiety, pain, and learning circuits (*24*, *36*) and extend it to demonstrate such heterogeneity in the context of arousal and wake-promoting circuits.

What underlies the specific fast surge of NE activity in the PRN? For long, a distinction has been made between tonic (slow) and phasic (fast) LC-NE activity patterns (*42*). Tonic firing relates to slow dynamics, internal states and arousal, such as vigilance states (*52*) and dynamics in the order of seconds or tens of seconds occurring during wakefulness (*53*, *54*) and NREM sleep rhythm (*11*, *43*). Phasic firing relates to sensory responses, decision-making, and network switching (*15*, *42*, *55*, *56*). A recent whole-brain optogenetics–functional magnetic resonance imaging study in mice revealed that LC surges are more likely to recruit regions involved in sensory processing (*51*). It is therefore likely that the PRN-NE surge we observe following sound presentation during sleep represents phasic LC firing in response to salient surprising stimuli. Phasic activity may express more strongly in adjacent brainstem regions as unmyelinated LC axons serve as a low-pass filter for distant NE signaling. Last, the findings indicate that SEAs are more effective in revealing LC-NE heterogeneity than spontaneous awakenings. This could possibly be related to sensory-evoked phasic activity rather than changes in tonic activity levels around spontaneous state transitions. Our results indicate that while both pathways reflect tonic-like state-dependent changes, the PRN is unique in displaying the phasic-like sensory-evoked response. PRN GRAB_NE_ signals during trials associated with awakenings are largely compatible with neuronal activities in *PRN-projecting LC*. Thus, these results suggest that the NE surge in the PRN is, at least partially, driven by *LC → PRN* neuronal activity, which may indeed reflect phasic firing.

A recent study showed that the PRN is involved in an auditory pathway implicated in SEA (*47*). The PRN receives input from the cochlear nucleus and has downstream targets involved in auditory responses during both NREM sleep and wakefulness (*48*). Our results further support this notion and highlight that, in addition to ascending auditory signaling via glutamatergic pathways, NE modulation at the PRN supports its key role in integrating arousal information into sensory pathways. Most of the PRN auditory-responsive neurons we recorded seem to receive monosynaptic input from the LC (fig. S7). Future research could characterize LC-NE PRN sensory tuning and wake-related responses beyond auditory modalities.

Some limitations in the current study could be overcome in future studies using refinements in research methodologies. One set of limitations concerns optogenetic silencing. At present, our silencing of projection-specific LC pathways with PdCO only provides temporal resolutions of many seconds. Thus, it was not possible to precisely time this intervention together with short auditory stimulation. Future studies could harness projection-specific silencing tools with higher temporal precision to test the effects of silencing *LC → PRN* at the specific windows of the early 0- to 1-s surge. In addition, bilateral silencing in PRN target regions is challenging due to the proximity of fiber-optics on the mouse brain. Future studies could explore such bilateral interventions, which have the potential of eliciting stronger effects.

A second domain of limitation concerns the interpretation of GRAB signals. The signal drop in both GRAB_NE_ and GRAB_MUT_ signals in the PRN ~1.5 s after sound onset remains poorly understood. In the GRAB_NE_ signal, this drop was significantly correlated with the preceding surge. Although our data cannot confirm a specific interpretation, one possibility is that an early NE surge in PRN leads to additional physiological process (for example: change in pH or in local blood perfusion) that affects both the GRAB_NE_ and GRAB_MUT_ signals. If so, this negative component, although not directly reflecting extracellular NE, may not be entirely artifactual either, in the sense that it could be related to some physiological process. While the physiological relevance of this signal drop remains unclear, its presence in the control sensor highlights an important caveat for interpreting NE sensor signals during state transition and underscores the need for caution and further methodological refinement when using GRAB sensors to study arousal-related processes. A third limitation concerns the interpretation of subpopulation recordings. Fiber photometry recordings of LC projection-based subpopulation may be less robust than full LC recordings, rendering the comparison between GCaMP7s and GRAB_NE_ signals difficult. Differences between signals could either reflect genuine differences in neuronal activity versus synaptic release or reduced GCAMP7s signal quality in subpopulation recordings. The constant improvements in recording methods and calcium indicators should allow better future monitoring of LC subpopulation activities based on projection targets. A fourth aspect of limitation is our focus on specific sensory modalities and brain regions. Our investigation focused on the auditory modality, building on previous work associating SEA with LC-NE activity (*17*). Future studies could also test sensory-evoked awakening along other modalities (e.g., somatosensation and olfaction) and seek to generalize findings to females. Last, the current study focused on the PRN and the BF as representing target regions in the brainstem, and forebrain, respectively. Future work can explore additional LC projection pathways known to be involved in arousal, such as the thalamus (*9*, *57*) and the hypothalamus (*58*).

In conclusion, our findings reveal that an early surge of NE signaling in the *LC → PRN* pathway contributes significantly to triggering awakenings in response to sounds, while the *LC → BF* pathway exhibits a slower, more delayed dynamic that may support sustained arousal. Although both pathways play important roles in SEA, the temporal distinction in their activation underscores functional specialization within the LC-NE system. More broadly, these results highlight the modular organization of LC outputs and support the growing view that distinct LC projection pathways mediate arousal and other behavioral functions in a target-specific manner. This work provides a framework for further dissecting how LC heterogeneity supports complex brain-state transitions.

## MATERIALS AND METHODS

### Animals

All experimental procedures including animal handling, surgery, and experiments followed the National Institutes of Health (NIH) Guide for the Care and Use of Laboratory Animals and were approved by the Institutional Animal Care and Use Committee of Tel Aviv University (approval 01-19-037, 01-22-004). Adult C57BL/6J (RRID:MGI:3028467) mice (8 to 12 weeks old at the time of surgery) were used for retrobeads and GRAB_NE_ experiments. Adult DBH-Cre mice were used for all other experiments. The mouse strain used for this research project, B6.FVB(Cg)-Tg(Dbh-cre)KH212Gsat/Mmucd, RRID:MMRRC_036778-UCD, was obtained from the Mutant Mouse Resource and Research Center (MMRRC) at University of California at Davis, an NIH-funded strain repository, and was donated to the MMRRC by MMRRC at University of California, Davis. This was made from the original strain (MMRRC:032081) donated by N. Heintz, Rockefeller University, GENSAT, and C. Gerfen, NIH, National Institute of Mental Health (*59*, *60*). For viral tracing, four mice were female, and one was male; all other experiments were performed on male mice. Mice were housed in transparent Plexiglas cages at constant temperature (20° to 23°C), humidity (40 to 70%), and circadian cycle (12-hour light/dark cycle, starting at 10:00 a.m.). Food and water were available ad libitum.

### Surgery

Before all surgical procedures, mice were anesthetized (with isoflurane 3% by volume for induction and 1.3% for maintenance) and placed in a stereotaxic frame. Surgery was performed under aseptic conditions, and the mice received antibiotics (cefazolin 15 mg/kg subcutaneously) and carprofen/Rimadyl 5 mg/kg intraperitoneally. For virus injections and fiber-optic implantations, a craniotomy was performed under microscopic control using a high-speed surgical drill. Injections were made using a UMP3 microsyringe injector and a Micro4 controller pump, and 33-gauge NanoFil needles (World Precision Instruments, USA) at a 100 nl/min pace. At the end of the injection, the scalp was sutured and the mouse returned to his home cage for 48 to 72 hours to allow for retrobead tracing and 3 weeks for retroviral tracing. The coordinates used to target BF were as follows: (respective to bregma) AP: 0.7 mm; mediolateral (ML): 2.25 mm at 7.5°; and DV: −5 mm relative to the brain surface. The coordinates used to target PRN were as follows: (respective to bregma) AP: −4.25 mm; ML: 1 mm; and DV: −4.8 mm relative to brain surface. Coordinates used to target the LC were as follows: (respective to bregma) AP: −5.4 mm; ML: 1 mm; and DV: −3.15 mm relative to brain surface.

For anatomical tracing, red fluorescent latex microspheres (retrobeads; Lumafluor) were used as a general retrograde tracker in a total volume of 300 nl injected into PRN or BF. Cre-dependent AAVretro vectors [ssAAV-retro/2-hSyn1-dlox-EGFP/mCherry(rev)-dlox-WPRE-hGHp(A)] were used as a specific noradrenergic retrograde neuronal tracer and injected into both regions—EGFP (enhanced green fluorescent protein) to the PRN and mCherry to the BF in a total volume of 500 nl for each injection (Fig. 1, A and E). For simultaneous GRAB_NE_ experiments, after injection of GRAB_NE_ virus [AAV2-hSyn1-GRAB(NE1m)-WRPE-hGHp(A); (*37*)] to PRN and BF, fiber-optics (MFC_400/430-0.48_5mm_MF1.25_FLT, Doric Lenses) were lowered to the same coordinates 0.15 mm higher in the DV axis (Fig. 2A). For retro ChR2 experiments, a Cre-dependent AAVretro with ChR2 [ssAAV-retro/2-hEF1α-dlox-hChR2(H134R)_mCherry(rev)-dlox-WPRE-hGHp(A)] was injected to either the PRN or the BF (as described above), and an fiber-optic was placed above the LC (MFC_200/240-0.22_5mm_MF1.25_FLT, Doric Lenses; Fig. 3, A and B). For control experiments, a fluorophore-only virus was used [ssAAV-retro/2-hSyn1-dlox-mCherry(rev)-dlox-WPRE-hGHp(A); fig. S4]. For PdCO experiments, the LC was injected with a Cre-dependent AAV with PdCO [ssAAV-1/2-hSyn1-lox71-ePdCO_mScarlet(rev)-lox66-WPRE-hGHp(A); (*41*)] or fluorophore only [ssAAV-5-hSyn1-dlox-mCherry(rev)-dlox-WPRE-hGHp(A), for control experiment; fig. S6] as described above. Three to 5 weeks after injection, fiber-optics (MFC_200/240-0.22_5mm_MF1.25_FLT, Doric Lenses) were implanted above PRN and BF (Fig. 4A).

At the end of all fiber-optic implantation surgeries, dental acrylic was gently placed around the fiber-optic, fixing them to the skull. Two screws (one frontal and one parietal; 1 mm in diameter), were placed over the left hemisphere for the EEG recording, and two additional screws were placed above the cerebellum and posterior parietal lobe as reference and ground (Fig. 4A). EMG was measured via two single-stranded stainless-steel wires inserted to either side of the neck muscles in a bipolar reference configuration. EEG and EMG wires were soldered onto a custom-made omnetics headstage connector. Metabond dental cement was used to cover all screws and EEG/EMG wires.

### Recording systems

#### *EEG*, *EMG*, *and video recordings*

EEG and EMG were digitally sampled at 1017 Hz [PZ5 amplifier, Tucker-Davis Technologies (TDT)] and filtered online: Both EEG and EMG signals were notch-filtered at 50 and 100 Hz to remove line noise and harmonics; then, the EEG and EMG signals were band-pass filtered at 0.5 to 200 Hz and 10 to 100 Hz, respectively. Simultaneous video data (for sleep and basic behavioral assessments) during free behavior were captured by a USB webcam synchronized with electrophysiology/photometry data.

#### Fiber photometry

Fiber photometry data were collected as described in (*61*). Briefly, using a one-site fiber photometry system (Doric Lenses, Canada) adapted to two excitation light-emitting diodes (LEDs) at 465 nm (GCaMP and GRAB_NE_) and 405 nm (isosbestic control channel). Simultaneous monitoring of the two channels was made possible by connecting the LEDs to a four-port minicube (with dichroic mirrors and cleanup filters to match the excitation and emission spectra; FMC4 or iFMC4, Doric Lenses, Canada) via an attenuating patch cord [400-μm core, numerical aperture (NA) = 0.37 to 0.48]. LEDs were controlled by drivers that sinusoidally modulated 465/405 nm excitation at 217/330 Hz, respectively, enabling lock-in demodulation of the signal (Doric Lenses, Canada). Zirconia sleeves were used to attach the fiber-optic patch cord to the animal’s cannula. Data were collected using Femtowatt Photoreceiver 2151 (Newport) or through an integrated photodetector in the case of iFMC4 and demodulated and processed using an RZ2 BioAmp Processor unit and Synapse software (TDT). The signal, originally sampled at 24,414 Hz, was demodulated online by the lock-in amplifier implemented in the processor, sampled at 1017.25 Hz and low-pass filtered with a corner frequency at 6 Hz. All signals were collected using Synapse software (TDT). To achieve 12/24-hour recordings with minimal adverse effects of prolonged LED activation (e.g., phototoxicity and photobleaching), we automatically turned the LEDs off every hour and allowed an hour with LEDs off (fig. S2).

#### Laser parameters for optogenetic experiments

Blue stimulation at 447 nm for optogenetic excitation and silencing was delivered via lasers (CNI, China) coupled to fiber-optics whose timing and intensity were automatically controlled via RZ2 (TDT). Light intensity at fiber-optic tips was measured with a power meter (Thorlabs PM100D) before fiber-optic insertion and set to 10 mW.

#### Auditory stimulation parameters

Sounds were generated in TDT software and amplified (SA1, TDT) and played free field through a magnetic speaker (MF1, TDT). Broadband noise (BBN) bursts of 1-s duration (0.8 V peak-to-peak waveform), in either 73, 80, or 88 dB sound pressure level (SPL), order randomized (27.94 ± 3.6%, 34.44 ± 4.34%, and 38.16 ± 4.26% awakening, respectively), were presented intermittently (±0.5-s jitter). Sound intensities were measured by placing a Velleman DVM805 Mini Sound Level Meter at the center of the cage floor.

### Experiments

At least 1 week of habituation was allowed between surgeries and experiments, after which, mice were placed in a home cage within a sound-attenuation chamber (−55 dB, H.N.A.) and connected to the EEG/EMG headstage and to the fiber-optic patch cord (MFP_400/430/1100-0.57_1m_FC-ZF1.25_LAF, Doric Lenses) for photometry recordings and (MFP_200/240/900-0.22_1m_FC-MF1.25, Doric Lenses) for optogenetic manipulations. A Logitech USB camera and MF1 magnetic speaker (TDT) were mounted 50 cm above the cage floor (Fig. 2A). After >48 hours in the new cage, habituation also gradually introduced tethering and exposure to sounds.

#### Optogenetics awakenings

Laser was triggered manually by the experimenter when the mouse was in NREM sleep (as observed using EEG, EMG, and video feed). Laser parameters were set to 10-s duration, 10-Hz frequency, and 90-ms duty cycle (fig. S1, E and F).

#### Undisturbed sleep

Electrophysiology and photometry data were recorded continuously for 12 hours (starting around light onset at 10 a.m.) while the mice were behaving freely (fig. S2 and Fig. 2C).

#### Sound-evoked arousal

BBNs as described above were presented intermittently in randomized order every 60 s (±0.5-s jitter) (Figs. 2, D to J, 3, F to H, 4, H and I, and figs. S3 and S6C).

#### Laser awakening experiment

Laser parameters for awakening experiments were 10 s duration and frequencies of 1, 5, 10, 20, and 40 Hz. Each parameter ran 10 times. The order of trials was randomized. Trial start was initiated manually so that all trials occurred during NREM after at least 15 s of continuous NREM with at least 30 s between trials (Fig. 3, C to E, and fig. S4).

#### Timing SEA experiment

To determine the criticality of synchronization between LC subpopulation activity and auditory stimulation, we changed the laser activation timing relative to sound. Laser parameters were 1 s and 20 Hz. Sound parameters for BBN bursts were 1-s duration at 80 dB SPL. Every 60 s (±0.5-s jitter), a trial was randomly initiated in which either laser started 1 s before sound, in time with sound, 1 s after sound, or 2 s after sound onset (Fig. 3, F to H).

#### PdCO experiments

The laser (447 nm) was set to 10 mW at fiber tip, 10-s stimulation, 20 Hz, 20 ms (Fig. 4).

##### Laser-only experiment

After habituation, mice were recorded, and stimulation was applied every 2 min (with 0.5-s jitter). Stimulation was either in the BF, the PRN, or both. Sham onsets were randomly selected from the data so that there were the same number of trials as in both site stimulation, and the times were between 50 and 70 s from other trial onsets (Fig. 4, C to G).

##### SEA + laser experiments

Stimulation was applied every 2 min (with 0.5-s jitter). Stimulation was either in the BF, the PRN, both, or none. At *time* = 8 s from laser onset, 1-s BBN was played (80 dB SPL) (Fig. 4, H and I).

#### Optotagging

For acute optoexcitation recording, 3 weeks after viral vector injection, under 0.9% isoflurane anesthesia, an optrode was lowered progressively in the PRN using the previously stated coordinates {A1x16-5mm-50-177-OZ16LP microelectrode PCB for scientific research, coupled with Optogenix tapered fiber [specifications: thickness (internal diameter/outer diameter) of 200/220 μm, NA = 0.39, active emitting length of 0.5 mm, termination at offset L1, tip of fiber at 250 μm above tip of the shank]; NeuroNexus, USA}. Ground and reference screws were placed in the frontal parts of the skull and tilted to the sides to minimize the danger to the shank. The optrode was connected to a PZ2 TDT amplifier through a ZIF-clip headstage. The fiber-optic was connected to a 477-nm laser that was set before the procedure to 10 mW at fiber tip. An MF1 speaker (TDT) was connected through a silicone tube to the contralateral hollow ear-bar. During the experiments, three BBN sounds were produced using Synapse (TDT) and were verified to match the sound intensities used in behavioral experiments. One-second BBNs at the three intensities were played in a random order, so that each sound was played 20 times. In addition, optogenetic stimulation was randomly spread throughout the experiment, so there were 20 laser stimulations of 5 s, at 10 Hz. At the end of the experiment, mice were euthanized and histologically verified before inclusion in the analysis (fig. S7).

### Histology

Following all experiments, under deep isoflurane anesthesia (4%) combined with a ketamine-xylazine dose (ketamine 100 mg/kg, xylazine 1.33 mg/kg), the mice were perfused intracardially with saline (0.9% NaCl; 1 ml/g) followed by 4% paraformaldehyde (PFA; Merck). Their brains were then extracted and fixed for 24 to 48 hours in 4% PFA. Coronal brain sections were cut using a Leica VT1000 S vibrating blade microtome at 60 μm. Then, sections were either kept free floating in phosphate-buffered saline (PBS) for immunohistochemistry or mounted on glass slides and examined under bright-field microscopy. Viral expression or retrobead localization was evaluated histologically by examination of double staining of free-floating sections. To achieve immunostaining, sections were washed three times in PBS (Hylabs) and then permeabilized in PBST (PBS containing 0.1% Triton X-100; Merck). Next, sections were blocked in PBST containing 20% normal goat serum (NGS; Vector Laboratories) for 1 hour at room temperature and incubated with primary antibodies in PBST (containing 2% NGS) at 4°C for 24 to 36 hours. After three washes in PBS, sections were incubated with secondary antibodies conjugated to fluorophores in PBST containing 2% NGS for 1.5 hours at room temperature. After three washes in PBST and once in PBS, the sections were mounted onto glass slides and coverslipped with an aqueous mounting medium (Thermo Fisher Scientific, catalog no. 9990412). Antibodies were against tyrosine hydroxylase to localize LC cells or red fluorescent protein (RFP) to improve PdCO viral expression visibility in axons [chicken anti–tyrosine hydroxylase (TH) 1:300, ab76442 Abcam, RRID: AB_1524535; guinea pig anti-RFP 1:500, 390004 SYSY, RRID:AB_2737052], and secondary antibodies conjugated to fluorophores were in appropriate coloring so as not to overlap with the viral fluorophore (AF405 goat anti-chicken 1:200, ab175674 Abcam, RRID:AB_2890171; AF488 goat anti-chicken 1:500, ab150173 Abcam, RRID:AB_2827653; AF488 goat anti-chicken 1:50, A11039 Invitrogen, RRID:AB_2534096; and donkey anti–guinea pig Cy5 1:300, 706-175-148 Jackson ImmunoResearch, RRID:AB_2340462). Images were acquired by a Leica SP8 high-resolution laser scanning confocal microscope (Leica, Wetzlar, Germany) and a ×10 air/0.4 NA objective, and contrast and brightness were improved for representative images.

### Statistical analysis

Unless stated otherwise, statistical analysis was done using analysis of variance (ANOVA) and Tukey-Kramer post hoc analysis. Shaded areas in graphs represent SEM. In box plots, the central mark in each box indicates the median, and the bottom and top edges of the box indicate the 25th and 75th percentiles, respectively. The whiskers extend to the most extreme data points not considered outliers. Dashed lines represent single animals. *P < 0.05; **P < 0.01; ***P < 0.001; ****P < 1 × 10^−4^. Illustrations were created using BioRender and Adobe Illustrator.

#### Fiber photometry

Data were detrended and normalized in periods when LED was active as described in (*11*). In short, the isosbestic channel was fitted to the GRAB trace using a linear polynomial fit, then used as the *f*_0_ reference point: $∆\frac{f}{f}=\frac{{\text{GRAB}}_{\text{NE}}-\text{Isosbestic}}{\text{Isosbestic}}\times 100\%$ (fig. S2).

#### Sleep scoring

Scoring of SEA was performed offline while visualizing EEG, EMG, and video data in TDT “scope” software so that the scorer was blinded to trial intensity. Each trial was given two scores: one characterizing the state at trial onset (wake/NREM/REM) and the other categorizing the behavioral outcome (maintained/awakening/EEG activation/EMG activation/short awakening). Baseline states were determined based on the state of the animal at the 5-s preceding trial. Cases in which the state was not stable at that time window were discarded. Behavioral categorization was determined based on the response from trial onset until 3 s later. Maintained sleep was declared only if there was no visible change in EEG, EMG, and video (38.14% of trials). Awakening was declared only in the case of EEG activation, EMG activation, and movement observed in the video that lasted at least 3 s (32.72% of trials; Fig. 2D). If all three conditions were observed for a period shorter than 3 s, the trial was tagged as “short awakening” (14.92% of trials). If only one was met, the trials were tagged as “EEG activation” (5.63%;) or “EMG activation” (8.59%) in accordance with the activated channel. Given the relatively lower probabilities and inconsistency of “semiawakening” trials (e.g., short, EEG, and EMG activation), those were eventually left out of analyses.

Undisturbed or laser-only recording sessions (without auditory stimulation) were scored continuously using semiautomatic sleep scoring as in (*62*) [adapted from (*63*)]. A >1-hour segment of manually scored data in each session was used in an automated sleep-scoring algorithm as in using a convolutional neural network that was trained using >20 manually scored blocks from prior mice recordings in the laboratory. The algorithm was fed with the manual sleep-scoring vector (tagged in 1-s resolution) along with the parietal EEG signal (low-pass filtered <20 Hz) and the EMG signal (band-pass filtered 10 to 50 Hz). The entire dataset was classified into sleep-scoring labels (wake/NREM/REM). Automatic classification of vigilance state was visually inspected to ensure accuracy (fig. S2E).

#### Analysis of anatomical profiles of LC → PRN and LC → BF subpopulations

##### Anatomical gradients of LC → PRN versus LC → BF subpopulations based on red fluorescent retrobeads data

For each animal, every coronal slice was localized to coordinates relative to bregma according to the Allen Mouse Brain Atlas, and its AP location was found (−5.34 to −5.8 mm). In every image, the LC dimensions based on TH immunostaining were manually marked. The image was divided into DV strips of 100 μm corresponding to −3.2 to −4.1 mm in the DV axes. Cells within each DV strip in every coronal slice of LC were manually counted by a blinded independent research assistant (data S1). Because every animal had different available coronal slices and the number of these available slices was varied, each cell count was weighted by the number of animals that contributed to it. Furthermore, to normalize the number of cells per animal, the count was divided by the average count for the same locations as well as the ratio between the animal yield and the expected yield. Then, the normalized values were multiplied by the average cell count to establish the cell proportion values (Fig. 1D).

Expected yield=Mean(#of cellsperstrip)

Animal yield=∑Cells in all strips peranimal

$$\begin{array}{c} \text{Normal yield}=\sum \text{Expected yield}\;\left(\text{in}\;\text{all}\;\text{locations that exist in current animal}\right) \end{array}$$

$$\begin{array}{c} \begin{array}{c} \text{NormalizedAnatomicalGradient}=\frac{\text{Cell count in every strip}}{\text{Expected yield}}/\frac{\text{Animal yield}}{\text{Normal yield}} \end{array} \end{array}$$

$$\text{averageDistribution}=\frac{\text{Expected yield}}{\sum \text{Expected yield}}$$

$$\begin{array}{c} \begin{array}{c} \begin{array}{c} \text{PRN}\;\text{distribution}=\left[\text{NormalizedAnatomicalGradient}\;\left(\text{in}\;\text{PRN}\;\text{animals}\right)\right]\\ \times \text{averageDistribution} \end{array} \end{array} \end{array}$$

$$\text{averagePRN distribution}=\frac{\text{PRN}\;\text{distribution}}{\sum \text{PRN}\;\text{distribution}}$$

$$\begin{array}{c} \text{BF}\;\text{distribution}=\left[\text{NormalizedAnatomicalGradient}\;\left(\text{in}\;\text{BF}\;\text{animals}\right)\right]\\ \times \text{averageDistribution} \end{array}$$

$$\text{averageBF distribution}=\frac{\text{BF}\;\text{distribution}}{\sum \text{BF}\;\text{distribution}}$$

$$\begin{array}{c} \text{PRN}-\text{BF}\;\text{diff prob}=\text{averagePRN distribution}-\text{averageBF distribution} \end{array}$$

For the weight by animal

$$\begin{array}{c} \text{freqValidAcrossAnimals}=\\ \text{Mean}\left(\text{cell sount}\;\text{per}\;\text{animal in}\;\text{all}\;\text{numeric values}\right) \end{array}$$

$$\begin{array}{c} \text{Mean absolute difference}=\\ \sqrt{\text{Mean}{\left(\text{PRN}-\text{BF}\;\text{diff prob}\right)}^{2}\times \text{freqValidAcrossAnimals}} \end{array}$$

Expected yields are shown in table S2, and the relative yield per animal is shown in table S3. To determine statistical significance, a Monte Carlo permutation test was used. First, the absolute difference between the two subpopulations was calculated as the square root of the mean square difference between the *PRN* and the *BF* distributions. Then, the same measurement was calculated on 100,000 randomly assigned permutations. The number of animals in each group was fixed, but the allocation of animals to every group was randomized. This created a distribution of possible mean absolute differences and allowed us to calculate the one-tailed *P* value associated under the hypothesis that the distributions are different. Upon determining a significant difference, we ran the same test to discover whether the difference was in the DV axis, the AP axis, or some combination. For each population, the mean location in each axis was calculated, and the difference between *PRN* and *BF* was used as the value to be compared. The resulting two-tailed *P* value was calculated from the distribution.

##### Colocalization of LC → PRN versus LC → BF neurons based on retrograde virus tracing

The double-labeling viral tracing quantification of the extent of colocalization of the red and green channels was carried out using Imaris Software (Imaris 9.0.1, RRID:SCR_007370). For each coronal slice, the LC boundaries were defined based on TH immunostaining. Cells in the two colors (red and green) within the LC were selected and were automatically counted using the “spots” tool, with a defined minimal radius of 15 μm and manually adjusted intensity. The number of colocalized cells was identified using the “colocalize spots” tool (with a maximum distance of 10 μm between the red and green spots). The position of each slice at the AP axis, relative to the Bregma, was manually matched to its corresponding position in the Allen Mouse Brain Atlas. For each slice, the ratio of colocalized cells to the total number of cells was calculated for both the BF and PRN projecting cells. The mean ratio of PRN and of BF colocalized cells per mouse was then obtained (Fig. 1, E to G, and table S4).

$$\begin{array}{c} \begin{array}{c} \text{BF}\;\text{ratio}\;\left(\text{per}\;\text{slice}\right)=\frac{\#\text{of}\;\text{BF}\;\text{and}\;\text{PRN}\;\text{cells}}{\text{Total}\#\text{of}\;\text{BF}\;\text{cells}}\;\\ \text{PRN}\;\text{ratio}\;\left(\text{per}\;\text{slice}\right)=\frac{\#\text{of}\;\text{PRN}\;\text{and}\;\text{BF}\;\text{cells}}{\text{Total}\#\text{of}\;\text{PRN}\;\text{cells}} \end{array} \end{array}$$

To determine statistical significance, two-sample *t* tests were applied to test whether each population is a subsample of the other. Thus, the mean ratio of colocalized cells per mouse for each subpopulation was tested against 1 (1 meaning all *BF* cells are also *PRN* cells or vice versa).

#### Analysis of simultaneous GRAB_NE_ data in PRN and BF and of retro-GCaMP

We examined average traces (across trials) of PRN and BF GRAB_NE_ after 5-s baseline subtraction (Fig. 2, C and E to J, and fig. S3B). To confirm that the baselines did not hold significant information, we compared the baselines under each outcome across mice using analysis of variance (one-way ANOVA) in PRN [*F*_3_ = 0.15, *P* = 0.93] and BF [*F*_3_ = 0.62, *P* = 0.6]. To determine temporal intervals associated with significant differences between NE dynamics in PRN and BF (horizontal bars at the bottom of Fig. 2F), we divided data to 0.3-s bins and compared the resulting 13 bins between *time* = 0 s and *time* = 4 s separately. Upon getting a significant Kruskal-Wallis test result [BF: $\chi^{2}\;$_13_ = 55.6, *P* = 3.17 × 10^−7^; PRN: $\chi^{2}\;$_13_ = 41.32, *P* = 8.45 × 10^−5^], we ran a one-sided multiple comparisons correction using Dunnett’s test to determine which bins were significantly above 0. Consecutive significant bins were grouped together, and the result was a significant time bin in the PRN trace: 0.3 to 0.6 s (PRN surge: *P* = 0.035), and a significant time bin in the BF trace: 1.8 to 3.9 s (BF rise: *P* = 0.015, 0.004, 0.005, and 0.01).

Data for spontaneous awakening from NREM sleep (Fig. 2C) were obtained from sessions in which no sounds were played, scored continuously (details above). Awakening trials were normalized to their −15: −10 s baseline.

#### EEG power analysis

EEG power analysis was performed on the parietal EEG channel using the “newtimef” function in MATLAB. Mean event-related (log) spectral perturbation (ERSP) was calculated on the trial matrix of −30 to 60 s around laser onset in each state (wake/NREM/REM) using FFTs and Hanning window tapering. The maximum window size is 10 s. The frequencies calculated from 1 to 50 Hz. Single trial normalization was not applied (Figs. 4F and 2J). The median ERSP over mice in each laser condition was calculated. Representational dissimilarity analysis was performed using the FieldTrip toolbox (*64*). In short, using one EEG channel, *t* tests were run comparing each condition with the sham condition. The final statistic was calculated using a Monte Carlo permutation test with cluster correction. Cluster alpha and configuration alpha were set to 0.1 for a one-sided test with a parametric cluster threshold. The number of randomizations was set to 1000.

EMG RMS was calculated over the trial (−5:15) EMG segment by binning to 100 ms and calculating for each bin the RMS. To account for variability across trials and animals, each trial was baseline subtracted. The 4 s from sound onset were averaged in each mouse.

#### State transition analysis

Awakening probability was calculated as the probability of full awakening over all NREM trials under the specific parameter (Figs. 3, E, F, H, and I, and 4J). Statistics were carried out using one-way ANOVA with Tukey-Kramer post hoc tests. For every PdCO mouse, the state it was in in each trial time point was added and divided by the number of trials that had the same base state at 5 s before trial onset. This resulted in a data structure containing the probability of each state from trial onset to 60 s after under the base state condition (Fig. 4D). Data were divided into dark and light phases, and after observing no discernable differences, only light phases results are displayed.

#### Spike sorting

Similar to (*65*), spike sorting was performed using “wave_clus” (*66*), using a detection threshold of 5 SD and automatic superparamagnetic clustering of wavelet coefficients. Clusters were manually selected and refined based on stability throughout recording, quality of separation from other clusters, consistency of spike waveforms, and interspike interval distributions as in (*67*).
