# Supplementary material for: An early surge of norepinephrine along brainstem pathways drives sensory-evoked awakening
Source: Sci Adv. 2025 Sep 10;11(37):eadw6375. doi: 10.1126/sciadv.adw6375 (PMC12422181; doi:10.1126/sciadv.adw6375)
Supplement: Supplementary file 1 — Figs. S1 to S7 Tables S1 to S6 Legend for data S1 [file sciadv.adw6375_sm.pdf]

Science Advances
AAAS

Supplementary Materials for

An early surge of norepinephrine along brainstem pathways drives sensory-evoked awakening

Noa Matosevich *et al.*

Corresponding author: Yuval Nir, ynir@tauex.tau.ac.il

Sci. Adv. **11**, eadw6375 (2025)
DOI: 10.1126/sciadv.adw6375

The PDF file includes:

Figs. S1 to S7
Tables S1 to S6
Legend for data S1

Other Supplementary Material for this manuscript includes the following:

Data S1

Supplementary Figures

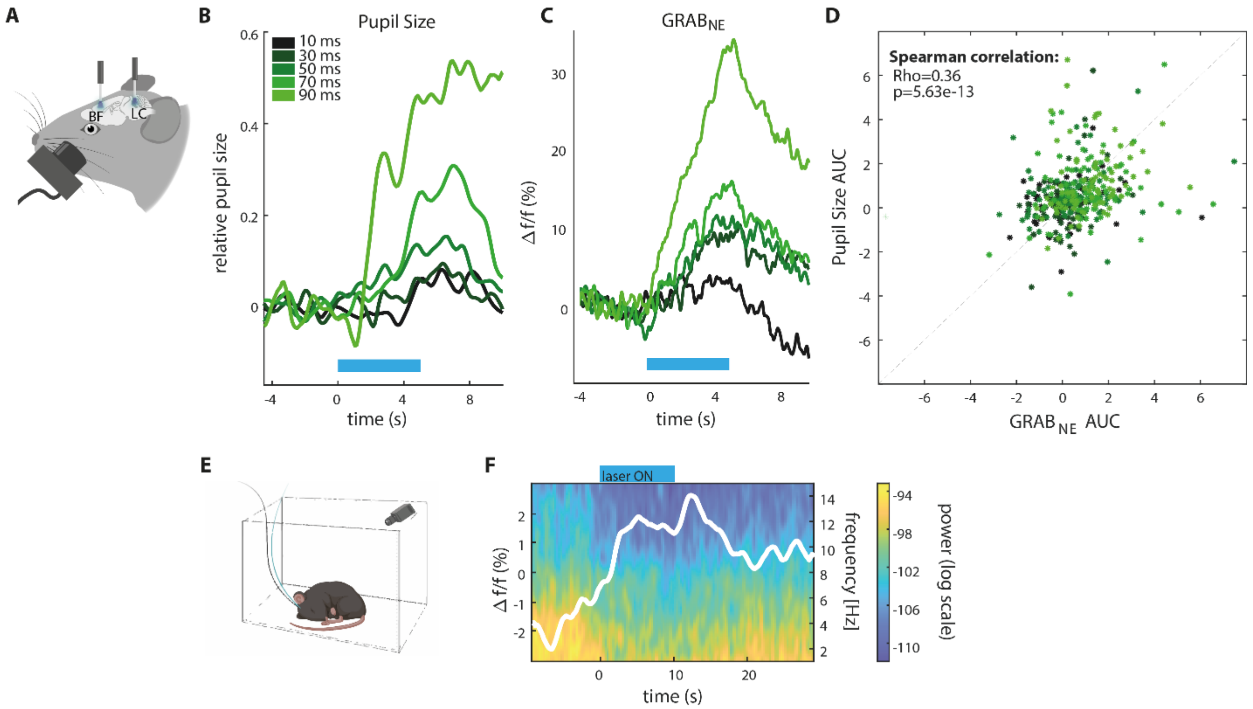

Figure S1: validating GRAB$_{NE}$ using optogenetics

A, Depiction of surgical approach and experimental procedure. The photometry fiber was placed above the BF, while the LC was injected with CAV2-PRS-ChR2-mCherry and an optic fiber was placed above it (MFC_200/240-0.22_5mm_MF1.25_FLT, doric). Mice (n=5) were anesthetized using isoflurane and placed in the recording chamber on a heating pad. A camera (Logitech C615) was focused on the ipsilateral eye, an infrared light was placed to allow capturing the pupil in dark conditions and optic patch cords were connected. Laser stimulations (10mW) were automatically triggered every 30 seconds, randomly iterating between parameters. Duration was set to 5 secs, frequency to 10 Hz, and duty cycle [10, 30, 50, 70 or 90 ms]. Simultaneous video data were captured by a USB webcam with the IR filter removed, synchronized with photometry data. To extract pupil area we applied the same pipeline as in (*17*, *41*), video images were first cropped around the eye. A mask based on the median values was applied and the best centrally fitted circle was selected using the "regionprops" function in Mathworks MATLAB. For each trial separately, pupil area was normalized by the average baseline pupil area in the 5 second preceding trial onset, and percent change dynamics were calculated for the [-5 30] seconds interval around stimulation. Trials were then averaged for each animal separately (12 to 20 trials per mouse, n=5) to generate time-courses. **B,** Representative example of pupil dilation as consequence of laser activation. Lines represent the average pupil traces per stimulation intensity (from dark to light green) in a single mouse. The Cerulean horizontal line represents laser activation (t=0-5s). **C,** Representative example of GRAB$_{NE}$ signal in the BF as consequence of laser activation. Lines represent the average pupil traces per stimulation intensity (from dark to light green) in a single mouse. The Cerulean horizontal line represents laser activation (t=0-5s). **D,** Correlation of pupil size and GRAB$_{NE}$ area under the curve (AUC). Dots are trials across all mice. Spearman Rho=0.36, p=5.63e-13. **E,** Depiction of experimental setup of awakening experiment. Created in BioRender. Regev, N. (2025) https://BioRender.com/2zvhz0k. **F,** Average spectrogram of EEG in a representative mouse across trials. White line represents average GRAB$_{NE}$ trace across trials. The Cerulean rectangle above represents laser activation (t=0-10 s).

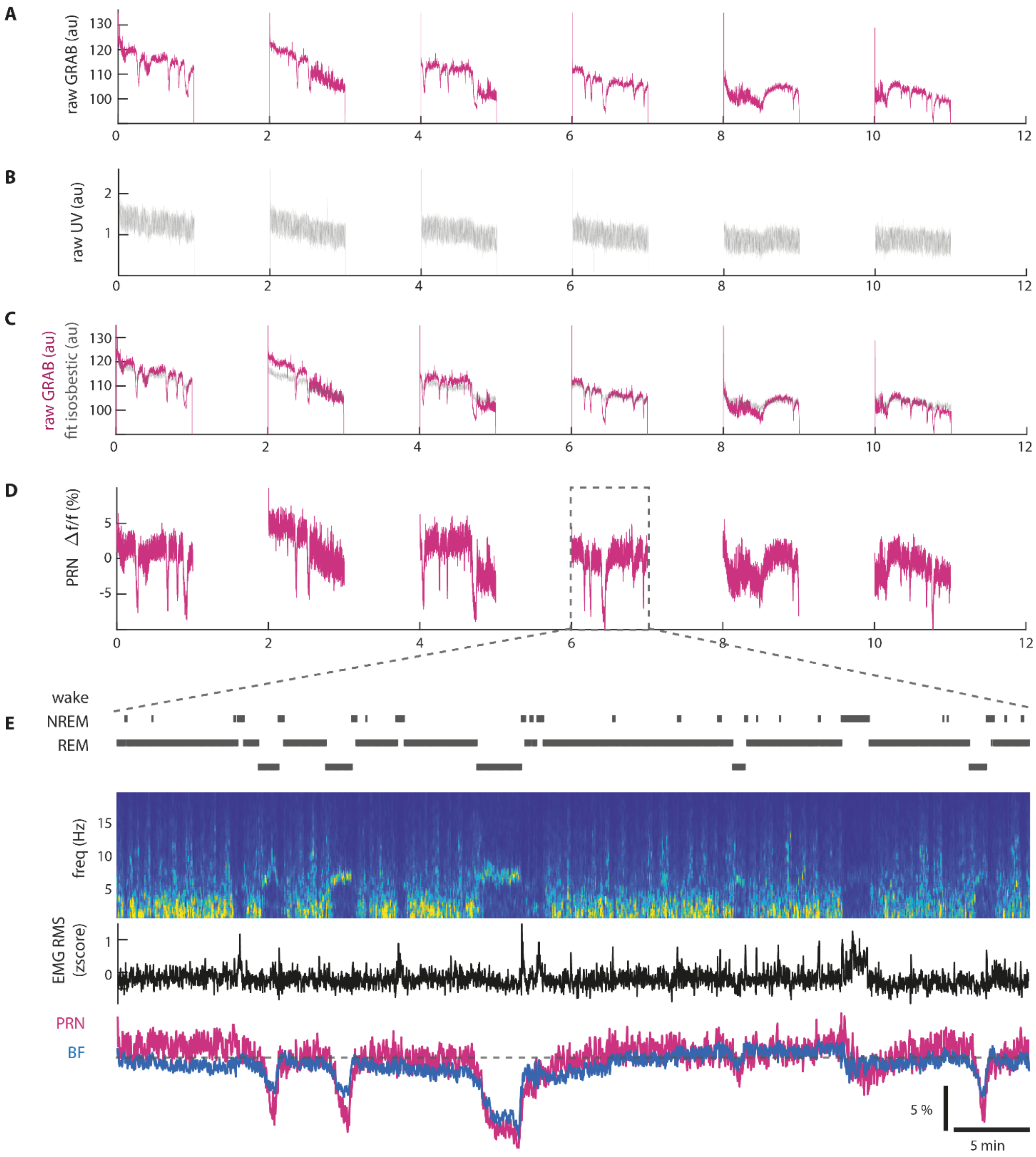

Figure S2: preprocessing and undisturbed sleep

A-D, Representative example of preprocessing pipeline, as in(*11*). **A,** Raw $GRAB_{NE}$ signal from PRN across a 12-hour recording. **B,** Raw isosbestic channel recording across 12 hours. **C**, Raw $GRAB_{NE}$ signal (magenta) overlaid with isosbestic channel after fitting (see methods). **D**, $\Delta f/f = \frac{GRAB - isosbestic}{isosbestoc}$ over 12 hours (see methods). **E**, One hour of full signal- from top to bottom: hypnogram, spectrogram of EEG, EMG root mean square, $GRAB_{NE}$ signal from PRN and BF in magenta and blue accordingly. Dashed line represents 0%. Scale bars represent 5%- and 5-min.

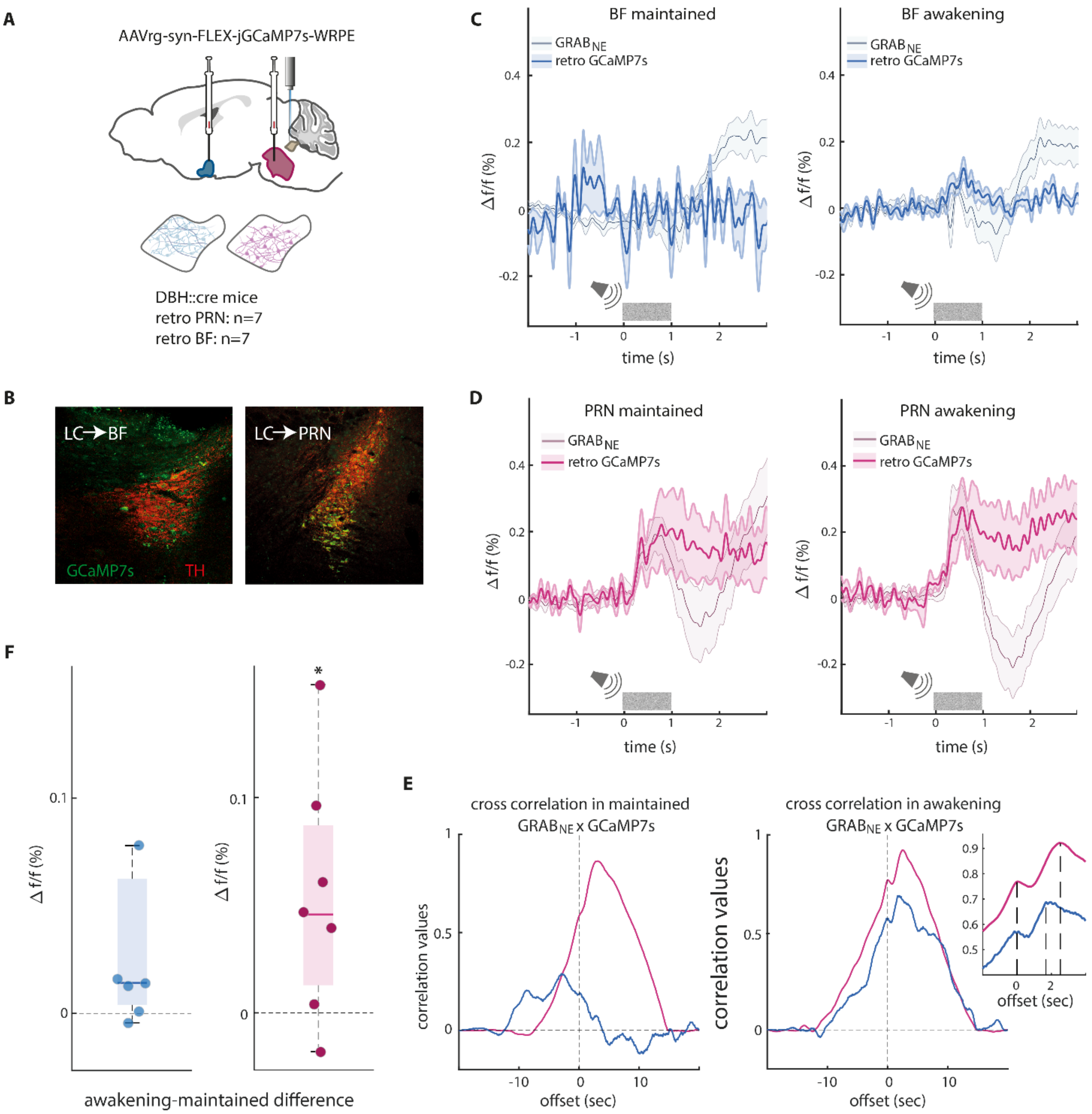

Figure S3: retro GCaMP

A, Depiction of surgical approach. A Cre-dependent retro AAV with GCaMP7s was injected to either PRN or BF (as described in Methods), and an optic fiber placed above the LC. **B**, Representative images of histology. **C,** Average GCaMP7s NREM SEA traces in LC→BF (blue), and the corresponding average trace in the $GRAB_{NE}$ recordings (gray), in maintained and awakening (left and right correspondingly) across mice (n=7). **D**, Mean ± SEM trace of LC→PRN (magenta) and the corresponding average trace in the $GRAB_{NE}$ recordings (maroon), in maintained and awakening (left and right correspondingly) across mice (n=7). **E,** Cross correlation between $GRAB_{NE}$ and retro GCaMP from the appropriate target (BF or PRN in blue and magenta correspondingly). Left- cross correlations in maintained trials, right- cross correlation in awakening trials. The average traces across mice were used for the cross correlation. For awakening correlations we added an inset zooming in to t=-2 to t=4, in order to clarify peak times. **F,** Box plot of awakening-maintained response differences (t=0-3s) in LC→BF and LC→PRN. Data points represent individual mice. $t_{PRN}(6)=2.52$, $p=0.045$; $t_{BF}(6)=1.64$, $p=0.15$.

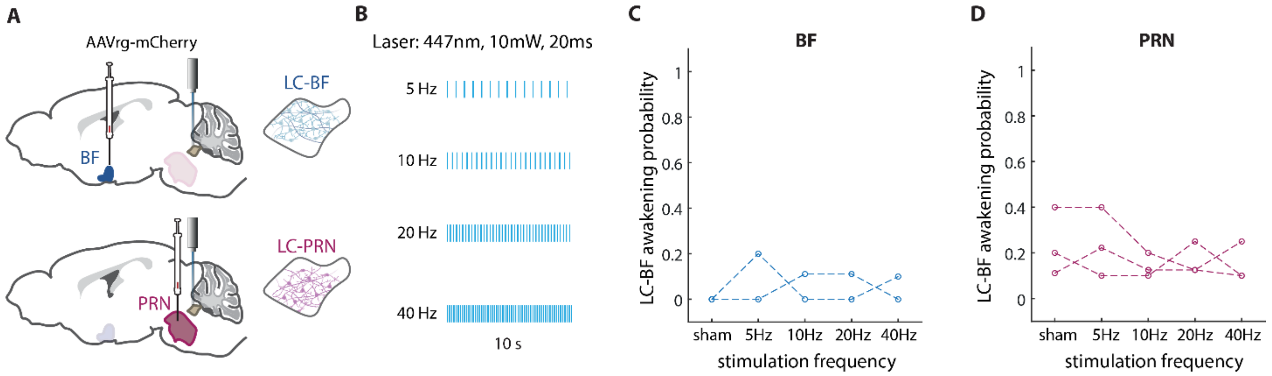

Figure S4: Fluorophore only control for opto-activation

A, Depiction of surgical approach for LC→BF (top), and LC→PRN (bottom). **B,** Experimental procedure for laser awakening experiment. The experiment included 10s laser of 10mW and 20ms duty cycle for 5Hz, 10Hz, 20Hz and 40Hz. **C, D,** Probability to awaken from laser activation as a function of frequency for LC→BF mCherry (**C**) and LC→PRN mCherry (**D**). Dots represent single animals (n_{BF}=2, n_{PRN}=3)

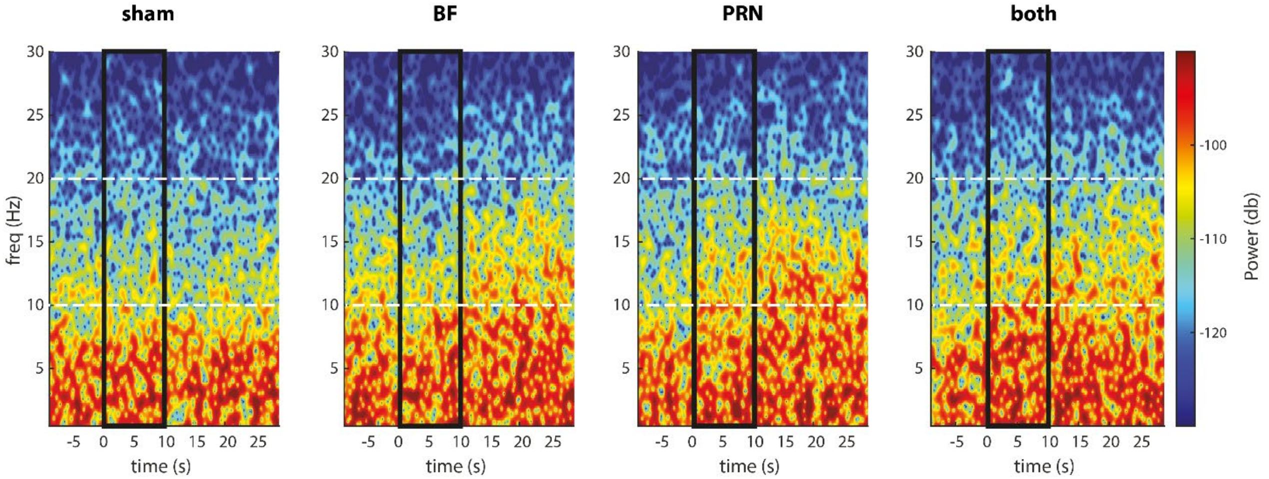

Figure S5: PdCO example spectrograms

Example spectrograms of EEG in undisturbed sleep under different laser activations (from left to right: sham, BF only, PRN only or both). Black squares mark the laser on times. White dashed lines delineate the 10-20 Hz range. In these examples we see that opto-silencing elevated power in the 10-20 Hz range, especially in the PRN and both conditions.

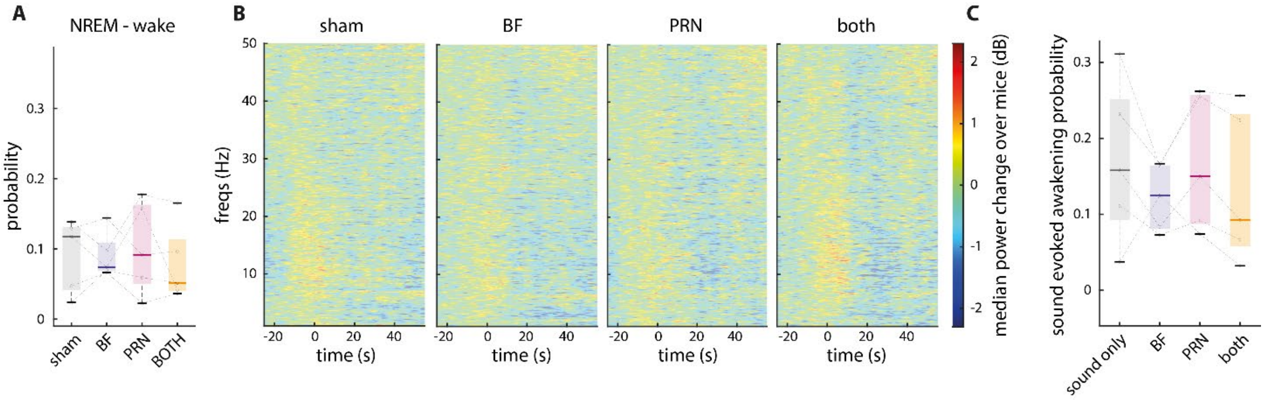

Figure S6: Fluorophore only control for selective synaptic silencing
A, Box plot of average probability to transition from NREM to wake under each condition during t=5-10 from laser onset. Dots represents single animals (n=5). **B**, Median EEG spectrograms across mice around trials during NREM normalized to t=-30-0s relative to laser onset. Laser was on from t=0 to t=10s. left- sham condition, BF laser condition, PRN laser condition, right- both lasers on condition. Areas that are not significant compared to sham are opaque. **C**, Box plot of awakening probability from SEA. Gray- sham, blue- BF, magenta- PRN, yellow-both. Dots represents single animals (n=5).

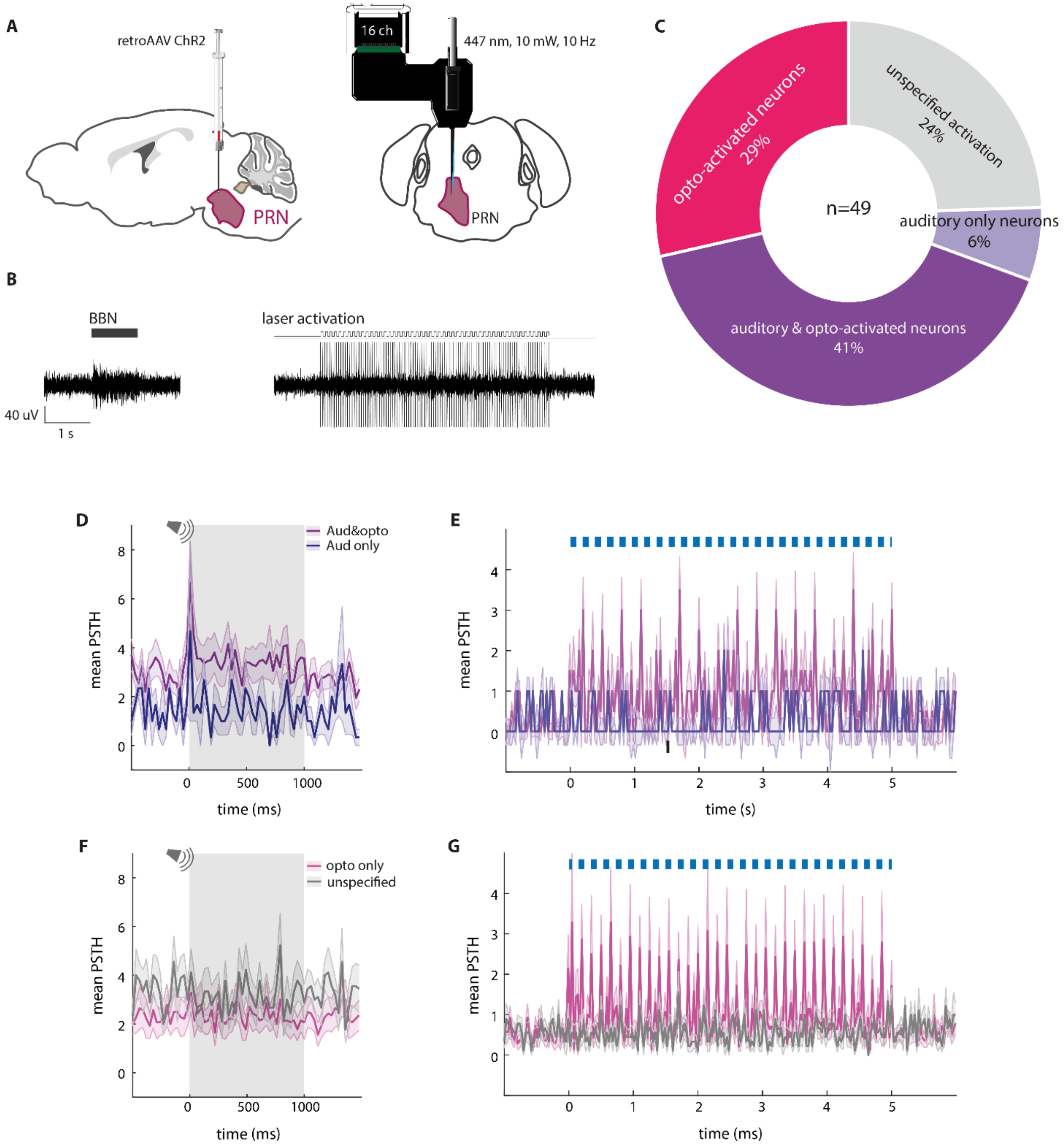

Figure S7: PRN electrophysiology. A, Left- Surgical procedure for injection of retroAAV to the PRN of DBH-cre mice. Right- Opto-electrode recording in the PRN. **B,** Example of high-pass filtered channel during BBN (left) and during laser activation (right). **C,** Quantification of unit identity. **D,** Mean PSTH during laser activation of neurons identified as auditory (divided to auditory-only units and to auditory and opto-responsive units in plum and magenta accordingly). **E,** Mean PSTH of opto-responsiveness in units identified as auditory (divided to auditory-only units and to auditory and opto-responsive units in plum and magenta accordingly). **F,** Mean PSTH during BBN of opto-responsive units and unidentified units in pink and gray accordingly. **G,** Mean PSTH of opto-responsiveness in units identified as opto-responsive and unidentified units in pink and gray accordingly.

Table S1- quantification of retrobead transfection in target regions

Specificity of transfection within ROI was calculated using ImageJ by marking the ROI in the corresponding coronal section in the mouse brain atlas and measuring the integrated density within the ROI divided by the total integrated intensity.

mouse	ROI	% within ROI
1	PRN	78.84615385
2	PRN	73.33333333
3	PRN	53.68421053
4	PRN	95.38461538
5	PRN	66.66666667
6	PRN	90.40419313
7	PRN	50.04453589
8	BF	81.81818182
9	BF	100
10	BF	64.40677966
11	BF	83.33333333
12	BF	100
13	BF	59.85472155
14	BF	60.73277579

PRN beads within ROI- 72.6% ±15.9%; BF beads within ROI- 78.6%±16.1%.

Table S2- Expected yield: average number of cells containing retrobeads per strip

Coordinates relative to Bregma (mm)	**anterior/ posterior**	-5.34	-5.4	-5.52	-5.68	-5.8
**dorsal/ ventral**						
-3.2		1.3636	1.7273	1.5	1.8571	1
-3.3		4	6.18	5.8	6.4286	8
-3.4		6.1818	8.4545	7.6	4.5714	6
-3.5		5.7273	8.6364	7.2222	5	9
-3.6		4.9091	5.1818	5.4286	1.6667	0
-3.7		5	2.2727	1	0	2
-3.8		5.5714	1	1	NaN	1
-3.9		13	0	NaN	NaN	NaN
-4		17	NaN	NaN	NaN	NaN
-4.1		2	NaN	NaN	NaN	NaN

Table S3- yield per animal compared to expected yield

mouse	ROI	Animal yield/ expected yield
1	PRN	1.78
2	PRN	1.31
3	PRN	0.55
4	PRN	0.83
5	PRN	0.79
6	PRN	1.13
7	PRN	0.88
8	BF	0.46
9	BF	1.21
10	BF	1.31
11	BF	0.47
12	BF	1.25
13	BF	0.58
14	BF	0.90

Table S4- double labeling analysis

ID	PRN only	BF only	co-localized
1	0.48917246	0.22665233	0.28417521
2	0.31875	0.45	0.23125
3	0.25026455	0.4005291	0.34920635
4	0.21876903	0.39267066	0.38856032
5	0.50212885	0.34271709	0.15515406
**mean**	**0.35581698**	**0.36251383**	**0.28166919**
**SD**	**0.11873354**	**0.07597195**	**0.0831161**

Table of the automated Imaris count data. Presenting neurons that were uniquely PRN, uniquely BF, or labeled by both viruses.

Table S5- AAVretro-ChR2 transfection efficiency

ID	ROI	slice	count TH	count ChR2	%	mean per animal
1	BF	1	14435	2079	14.40249	14.76728
		2	16204	2452	15.13207	
2	BF	1	12941	1677	12.95881	12.95881
3	BF	1	36616	3892	10.62923	9.447982
		2	43406	6508	14.99332	
		3	35717	972	2.721393	
4	BF	1	62209	10744	17.27081	23.45508
		2	50530	14294	28.28815	
		3	58207	14439	24.80629	
5	BF	1	43636	9575	21.94289	17.92134
		2	32965	2420	7.341119	
		3	34424	8427	24.48001	
6	BF	1	4740	914	19.2827	19.2827
7	PRN	1	27298	3827	14.01934	21.96691
		2	38590	11544	29.91449	
8	PRN	1	45297	8403	18.5509	21.20422
		2	40067	9559	23.85754	
9	PRN	1	45618	4768	10.45201	11.09253
		2	52220	6127	11.73305	
10	PRN	1	29753	2027	6.812758	6.812758
11	PRN	1	24197	3239	13.38596	13.38596

The table consists of the pixel count acquired using ImageJ software. 'Count TH' represents the pixel count of TH labeled cells. 'Count ChR2' represents the number of pixels with the red mCherry fluorophore that was expressed as part of the AAVretro virus. The percentage of ChR2 from TH+ was calculated, then averaged within each animal.

Table S6- multinomial regression model of single trial behavioral outcome

	Value	SE	tStat	pValue
**Intercept awakening**	**0.25**	**0.08**	**3.32**	**9.03 e-04**
**PRN surge awakening**	**-0.55**	**0.13**	**-4.11**	**4 e-05**
BF rise awakening	0.02	0.19	0.1	0.92

Supplementary data 1- retrobead neuronal count

The excel file has the full neuronal count data. Every tab is a single mouse, and every cell represents the number of TH+retrobead neurons were manually counted in the corresponding strip. NA means that the strip does not exist, either there was no corresponding section for the mouse, or the strip is outside the bounds of the LC, as identified by TH labeling.
